# Highly motile cells are metabolically responsive to collagen density

**DOI:** 10.1073/pnas.2114672119

**Published:** 2022-04-26

**Authors:** Matthew R. Zanotelli, Jian Zhang, Ismael Ortiz, Wenjun Wang, Neil C. Chada, Cynthia A. Reinhart-King

**Affiliations:** ^a^Nancy E. and Peter C. Meinig School of Biomedical Engineering, Cornell University, Ithaca, NY 14853;; ^b^Department of Biomedical Engineering, Vanderbilt University, Nashville, TN 37235

**Keywords:** cell migration, mechanobiology, extracellular matrix, metabolism, heterogeneity

## Abstract

Altered tissue mechanics and metabolism have gained significant attention as drivers of tumorigenesis, and mechanoresponsive metabolism has been implicated in migration and metastasis. However, heterogeneity in cell populations makes it difficult to link changes in behavior with metabolism, as individual cell behaviors are not necessarily reflected in population-based measurements. As such, the impact of increased collagen deposition, a tumor-associated collagen signature, on metabolism remains ambiguous. Here, we utilize a wide range of collagen densities to alter migration ability and study the bioenergetics of individual cells over time. Sorting cells based on their level of motility revealed energetics are a function of collagen density only for highly motile cells, not the entire population or cells with low motility. Changes in migration with increasing collagen density were correlated with cellular energetics, where matrix conditions most permissive to migration required less energy usage during movement and migrated more efficiently. These findings reveal a link between matrix mechanics, migratory phenotype, and bioenergetics and suggest that energetic costs are determined by the extracellular matrix and influence cell motility.

Metabolic reprogramming is a fundamental aspect of cancer that contributes to transformation and tumorigenesis (1). Cancer cells exhibit altered metabolism compared to nonmalignant tissue as well as extensive metabolic heterogeneity resulting from factors intrinsic to cancer cells and microenvironmental cues (2). During tumor progression, loss of tissue homeostasis and aberrant tissue mechanics play a critical role in driving invasion and malignancy (3, 4), as mechanical cues have been linked to almost all the hallmarks of cancer including altered metabolism (5). In breast cancer, tumor-associated collagen signatures including increased collagen deposition have been identified and correlated with aggression and patient prognosis (4). However, metabolic changes in response to collagen density remain unclear. Some work indicates that dense matrices slowed metabolism (6), while others suggest increased density increased metabolic activity (7). We hypothesize that the differences reported are due to the heterogeneity within cell populations and important changes can be masked by population-averaged measurements. Cellular energetics are linked to migration speed (7) and the metabolic reprogramming of cancer cells impacts their metastatic potential (8, 9), suggesting metabolism impacts migration. Cancer cells phenotypically sorted based on invasion do demonstrate unique molecular programs including those related to metabolism (10). Here, we examined the metabolism of the cell population and bioenergetics of individual cells encapsulated in a wide range of collagen densities to manipulate migration difficulty. We find metabolic activity is correlated with the motile fraction of each density and only the highly motile subpopulation exhibit changes in intracellular adenosine 5′-triphosphate:adenosine 5′-diphosphate (ATP:ADP) ratio. Net migration and ATP:ADP are a function of collagen density, and the energy costs per unit of migration in each density are significantly correlated with the number of highly motile cells in each population. The single-cell measurements in this work indicate that changes in cell migration in response to matrix cues are associated with altered bioenergetics, where energy usage is associated with migration ability.

## Results

### Cell Motility Is Correlated with Metabolic Activity.

**Fig. 1.**
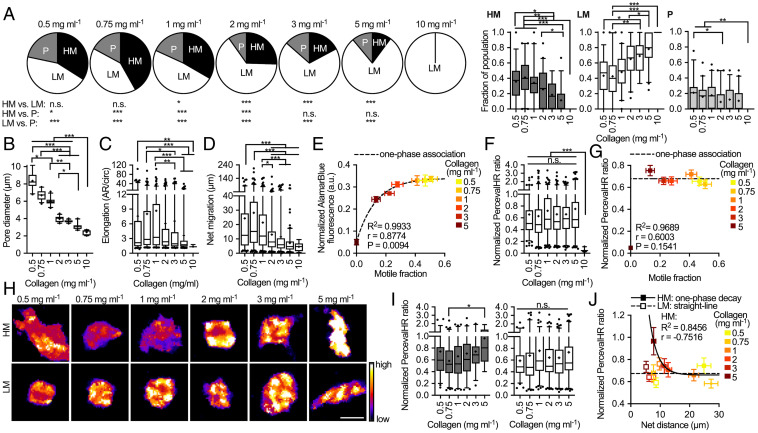
Migration activity influences metabolism with changes in collagen density. (*A*) Distribution of highly motile (HM), lowly motile (LM), and proliferating (P) cells with collagen density (*n* = 212, 251, 264, 224, 221, 226, and 78). (*B*) Pore diameter (*n* = 30), (*C*) cell elongation, (*D*) net migration, (*E*) normalized AlamarBlue fluorescence vs. motile fraction, (*F*) normalized PercevalHR ratio, and (*G*) normalized PercevalHR vs. motile fraction with collagen density (*n* = 126, 167, 167, 160, 149, 164, and 31). (*H*) ATP:ADP heat map, (*I*) normalized PercevalHR, and (*J*) normalized PercevalHR vs. net distance of HM (*n* = 55, 85, 78, 52, 33, and 16) and LM cells (*n* = 71, 82, 99, 115, 121, and 150). *P < 0.05, **P < 0.001, ***P < 0.0001, n.s. = not significant. (Scale bar, 15 μm.).

MDA-MB-231 cancer cells were encapsulated in 0.5 to 10 mg⋅mL^−1^ type I collagen and categorized based on their motility over 6 h [high motility (HM): migrated >15 μm in 2 h (11), low motility (LM), or proliferating (P)]. The fraction of HM cells changed as a function of collagen density, with motility highest in 0.75 mg⋅mL^−1^ (Fig. 1*A*). Pore size decreased with increasing density, while elongation, a measurement of cell spreading, increased then decreased with increasing density (12) (Fig. 1 *B* and *C*). Net migration demonstrated a relationship like motility fraction and migration was highest in 0.75 mg⋅mL^−1^ (Fig. 1*D*), suggesting an intermediate level of confinement and cell-substrate area is optimal for migration and high-density collagen restricts movement. AlamarBlue, which uses the reducing power of living cells to detect metabolically active cells, was then used to measure the metabolic activity in each density and positively correlated with motile fraction (Fig. 1*E*). These results indicate that migration, both number of migratory cells and distance traveled by individual cells, is associated with metabolic activity for each density of gel.

### The Bioenergetics of Highly Motile Cells Respond to Matrix Density.

Given metabolic activity of the cell population is associated with motility fraction, we compared the energetics of cells with high and low motility. Using MDA-MB-231 cells expressing the fluorescent intracellular ATP:ADP biosensor PercevalHR (13), we observed no change in ATP:ADP ratio with collagen density when assaying across the entire cell population except in 10 mg⋅mL^−1^, where migration stopped and ATP:ADP decreased, and there was no relationship between ATP:ADP and motile fraction (Fig. 1 *F* and *G*). However, the HM subpopulation exhibited increased ATP:ADP with increasing density and a significant difference between 0.75 and 5 mg⋅mL^−1^ (Fig. 1 *H* and *I*). Across collagen densities, the ATP:ADP of HM cells had a significant positive correlation with net migration, which was not observed in LM cells (Fig. 1*J*). These findings reveal migratory cells, but not nonmigratory cells, are metabolically responsive to collagen density.

### Migration Ability Is Associated with Energy Expenditure for Movement.

**Fig. 2.**
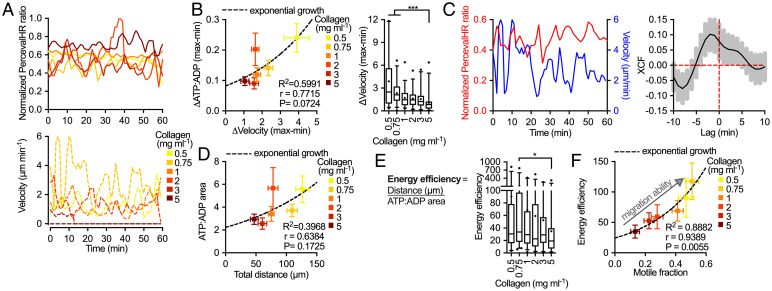
Energy costs during migration drive migration ability. (*A*) ATP:ADP and velocity fluctuations of individual cells and (*B*) ATP:ADP change vs. velocity change with collagen density. (*C*) ATP:ADP and velocity measurements in 0.75 mg⋅mL^−1^ and the cross-correlation factor (XCF) vs. time (*n* = 39). (*D*) ATP:ADP area vs. total distance across collagen densities. (*E*) Energy efficiency of migration in each collagen density and (*F*) its relationship with motile fraction (*n* = 28, 39, 38, 28, 27, and 32). *P < 0.05, ***P < 0.0001.

As migratory cells are metabolically sensitive to collagen density, we analyzed ATP:ADP fluctuations during migration (Fig. 2*A*). Energy and velocity fluctuations changed with density and conditions with the highest motility fraction demonstrated large ATP:ADP changes accompanied with significantly larger velocity changes (Fig. 2*B*). To better understand this relationship, we performed temporal cross-correlation (Fig. 2*C*). ATP:ADP and velocity signals were positively correlated and increased ATP:ADP was accompanied by increased velocity. Increased ATP:ADP is necessary for membrane reorganization (14) and migration through restricting spaces (13, 15), and these findings suggest ATP is used during movement and energy production is increased to meet demands. ATP:ADP area (total area under the ATP:ADP curve vs. time) was then used to assess energy usage of individual cells and was associated with total migration distance across densities (Fig. 2*D*). Energy efficiency of migration (total distance/ATP:ADP area) responded to increasing collagen density like motile fraction, with the most efficient migration in 0.75 mg⋅mL^−1^ and a significant difference between 0.75 and 5 mg⋅mL^−1^ (Fig. 2*E*). Energy efficiency was significantly correlated with motile fraction and reflected migration ability in each density (Fig. 2*F*). Together, these findings show collagen densities that support the highest motile fraction and are the most permissive to migration also allow cells to use less energy and travel more efficiently.

## Discussion

The mechanical microenvironment is a critical regulator of cell migration and tumorigenesis. Our previous work indicated that intracellular ATP:ADP ratio of individually migrating cells is correlated with glucose uptake, ATP hydrolysis, and velocity as a function of collagen density, where cells with higher ATP:ADP had higher velocities (7). However, other studies have suggested cells in high-density collagen exhibit decreased oxygen consumption and ATP production (6). We find that these differences are due to the heterogeneity within cell populations. While population-based analysis demonstrated no changes in cellular energetics, our data revealed that the HM subpopulation has notable differences in ATP:ADP and energy usage that are correlated with migration ability, suggesting motility is influenced by energy costs for migration. Matrix mechanics can impose high energetic costs to direct migration path (13), and mechanosignaling–metabolism cross-talk has been shown to support energy production during metastasis (16, 17). Stiff stroma triggers metabolic changes through YAP-driven mechanosignaling that facilitate rapid ATP production and recycling to support invasion (17). Similarly, thick actin bundles can also resist the degradation of glycolytic enzymes, which normally occurs in response to increased matrix stiffness, and maintain high energy production regardless of mechanical stimuli (16). Our findings that migration efficiency is impacted by collagen density suggest mechanosignaling may support high metabolic activity during invasion through challenging microenvironments. These data underscore the presence of phenotypic heterogeneities which are overlooked in population-averaged measurements.

## Materials and Methods

All experiments were done as previously described (13). Detailed descriptions are provided in *SI Appendix*.

## Supplementary Material

Supplementary File

## Data Availability

All study data are included in the article and/or *SI Appendix*.
