# Supplementary material for: Highly motile cells are metabolically responsive to collagen density
Source: Proc Natl Acad Sci U S A. 2022 Apr 26;119(18):e2114672119. doi: 10.1073/pnas.2114672119 (PMC9170068; doi:10.1073/pnas.2114672119)
Supplement: Supplementary File [file pnas.2114672119.sapp.pdf]

PNAS
www.pnas.org

Supplementary Information for
Highly motile cells are metabolically-responsive to collagen density

Matthew R. Zanotelli[1,2], Jian Zhang[2], Ismael Ortiz[2], Wenjun Wang[2], Neil C. Chada[2], Cynthia A. Reinhart-King[1,2*]

[1]Nancy E. and Peter C. Meinig School of Biomedical Engineering, Cornell University, Ithaca, NY 14853, USA

[2]Department of Biomedical Engineering, Vanderbilt University, Nashville, TN 37235, USA

*Corresponding author
Email: cynthia.reinhart-king@vanderbilt.edu

This PDF file includes:

Supplementary Materials and Methods

Supplementary Materials and Methods

Cell culture. MDA-MB-231 cells (HTB-26, ATCC) were maintained and transduced with PercevalHR and pHRed, as previously described (13).

Cell encapsulation. 10 mg ml^{-1} acid solubilized type I rat tail tendon (Pel-Freez Biologicals, Rogers, AR, USA) collagen was diluted using DMEM on ice and neutralized to pH 7.0 using 1N NaOH (13). Cells were added at 125,000 cells ml^{-1}, mixed, 500 μl added to a glass bottom 24-well plate (MatTek Corporation, Ashland, MA), then polymerized for 45 min at 37°C and overlaid with culture media.

Confocal reflectance microscopy. Collagen fiber structure was visualized using a Zeiss LSM 800 inverted confocal microscope equipped with a 640 nm laser using a 40x/1.1 N.A. long working distance water-immersion objective and operated by Zen 2.3 software.

Confocal microscopy. Imaging was performed using a Zeiss LSM 800 operated by Zen 2.3 software with a 20x/0.8 N.A. and 40x/1.1 N.A. water-immersion objective for PercevalHR/pHRed and reflectance, respectively (13).

Collagen architecture analysis. Pore size was quantified in MATLAB (Mathworks, 2015b) (12).

Cell morphology analysis. Cells features including aspect ratio and circularity were quantified in ImageJ using the measure tool to manually outline the cell body. Elongation (aspect ratio/circularity) was then calculated to determine changes in cell morphology.

Cell migration analysis. Cells were manually outlined over 12-18 h (20 min intervals) and 12-13 h (1 min intervals) to measure centroid and morphology. A cell had high motility if it traveled >15 μm, the approximate diameter of an MDA-MB-231 cell in suspension (11), in 2 h. Cells that divided or interacted with other cells were excluded from analysis.

AlamarBlue cell viability assay. AlamarBlue stock reagent (Invitrogen) was dissolved 1:10 in culture media and cells were incubated for 24 h. Fluorescent intensity was measured using a plate reader at 560/590 nm (ex/em). To account for changes in proliferation, AlamarBlue was normalized to total cell count:

$$Normalized\ AlamarBlue = \frac{AlamarBlue\ fluorescence\ intensity}{seeding\ density + (seeding\ density * \%proliferating)}$$

Quantification of intracellular ATP:ADP ratio. PercevalHR ratio (F488/F405) and pHRed ratio (F561/F488) were measured and normalized PercevalHR ratio was calculated using a customized ImageJ macro (version 2.0.0-rc-68/1.5g, National Institutes of Health), as previously described (13). From 1 h time-lapses, normalized PercevalHR and velocity were smoothed using LOWESS regression to reduce noise, then ATP:ADP and velocity fluctuations and ATP:ADP area (area between curve and lowest value using trapz function) was calculated in MATLAB (Mathworks, 2019a). Energy efficiency was calculated as total migration/ATP:ADP area. Temporal cross-correlation was done in MATLAB (R2019a, Mathworks) using the crosscorr function.

Image generation. Representative images of intracellular ATP:ADP ratio were generated as pixel-by-pixel ratio images and displayed as heatmaps using ImageJ. Adjustment of display map intensity, re-sizing, and addition of scale bars was all performed in ImageJ.

Statistical analysis. Statistical analysis was performed using GraphPad Prism 9.0, as previously described (13). Data shown as median ± interquartile range (box), 5th–95th percentiles (whiskers), and mean (+), or mean ± s.e.m. All experiments were reproduced at least three independent times.
